# Implications of Pulsed Electric Field Pre-Treatment on Goat Milk Pasteurization

**DOI:** 10.3390/foods12213913

**Published:** 2023-10-25

**Authors:** Alberta Araújo, Carla Barbosa, Manuel Rui Alves, Alexandre Romão, Paulo Fernandes

**Affiliations:** 1 CISAS, Escola Superior de Tecnologia e Gestão, Instituto Politécnico de Viana do Castelo, Rua Escola Industrial e Comercial de Nun’Álvares, 4900-347 Viana do Castelo, Portugal; alberta@estg.ipvc.pt (A.A.); mruialves@estg.ipvc.pt (M.R.A.); paulof@estg.ipvc.pt (P.F.); 2 CEB—Centre of Biological Engineering, Universidade do Minho, 4710-057 Braga, Portugal; 3 LAQV-Requimte, Associated Laboratory for Green Chemistry—Network of Chemistry and Technology, Faculdade de Farmácia da Universidade do Porto, 4050-313 Porto, Portugal; 4 Escola Superior de Tecnologia e Gestão, Instituto Politécnico de Viana do Castelo, Rua Escola Industrial e Comercial de Nun’Álvares, 4900-347 Viana do Castelo, Portugal; alexandre.m.romao@gmail.com

**Keywords:** pulsed electric fields, PEF, goat’s milk, cow’s milk, *Listeria monocytogenes*, *E. coli*, pasteurization

## Abstract

Goat milk is an interesting product from a nutritional and health standpoint, although its physico-chemical composition presents some technological challenges, mainly for being less stable than cow’s milk at high temperatures. As pasteurization and ultra-high temperature processing are universally employed to ensure milk quality and safety, non-thermal methods, such as pulsed electric fields (PEFs), reduce the microbial load and eliminate pathogens, representing an interesting alternative for processing this product. This study demonstrates how the combined use of a PEF with short thermal processing and moderate temperature can be effective and energy-efficient in goat milk processing. A combination of thermal treatment at 63 °C after a low-intensity PEF (50 µs pulses, 3 Hz, and 10 kV·cm^−1^) caused the same reduction effect on the population of *Listeria monocytogenes* (goat’s raw milk artificially spiked), as compared to a thermal treatment at 72 °C without a PEF. However, *z* values are significantly higher when PEF is used as a pre-treatment, suggesting that it may induce heat resistance in the survival population of *L. monocytogenes*. The sensitivity of *L. monocytogenes* to high temperatures is less pronounced in goat’s milk than cow’s milk, with a more pronounced impact of a PEF on lethality when combined with lower temperatures in goat’s milk. The effect of a PEF on *Escherichia coli* viability was even more pronounced. It was also observed that thermal treatment energy needs with a PEF as a pre-treatment can be reduced by at least 50% of the total energy requirements.

## 1. Introduction

According to statistics from 2018 to 2019, goats produce about 15.26 million tons of milk annually [1,2]. In Portugal, in 2021, goat’s milk accounted for only 1.46% of total milk production [3]. The unique composition and physico-chemical characteristics of goat’s milk [1,4,5,6,7] impact its behavior when subjected to processing. In fact, the heat stability of goat’s milk is lower than that of cow’s milk, and its coagulation pattern is highly dependent on initial pH [8], among other factors [9,10], thus being more susceptible to damage when treated with conventional thermal treatments [11]. The current and most common method used to maintain milk’s safety and shelf-life stability is pasteurization using a thermal treatment, which typically ranges from 72 °C to 80 °C or even higher temperatures in the case of UHT milk. These heating temperatures promote the denaturation of whey proteins and the aggregation of casein micelles, which are more severe in goat’s milk due to very small casein micelles and fat globules, leading to a gradual decrease in quality and acceptability of commercial goat milk [12,13,14]. The effect of high-temperature treatments on physical and organoleptic properties, like nutritional losses, color modifications, and flavor changes, has led to an exploration of emerging technologies to produce milk products with better retention of nutrients and fresh-like characteristics of milk components [15,16,17,18]. PEF technology is an emerging technology that has been considered an alternative to conventional pasteurization technology. However, although promising, the industry has not yet adopted this new technology, mostly due to a lack of information concerning investment needs and their scale-up viability [17,19].

PEF equipment generally comprises an electrical pulse-generating device, a chamber where the electric pulses trespass the food product, and circulating pumps. PEF technology consists of an application of high voltage (usually 20–50 kV·cm^−1^ and even higher) with short pulses (a few to tens of μs) at a pulse-defined frequency (Hz), which will increase temperature (°C) through liquid foods or liquid-immersed foods [19,20]. Thus, the electrical breakdown of the cell membrane will occur, acting as a capacitor filled with a dielectric medium. This is the mechanism by which microbes are inactivated using PEF treatment [21]. These parameters (pulse width and frequency, potential difference between electrodes, and flow rate in the case of continuous treatments) directly affect the viability of microorganisms [20], but the adverse effects of thermal processing are minimized, allowing the preservation of the natural fresh-like characteristics. Moreover, this technology offers other advantages, such as a low processing cost, higher energy efficiency, and environmental sustainability [22,23].

However, some authors state that the industry has shown some resistance to adopting this technology due to the high initial investment costs. Nevertheless, those studies are quite outdated, not taking into consideration the development of the PEF technology and the low maintenance costs [19]. According to Arshad et al. [22], PEF processing is an energy-conservation technique compared to thermal processing. These authors have also outlined that conventional pre-heaters in some food pre-treatments require higher energy than the energy obtained as a consequence of PEF pre-heating and offer an exclusive opportunity to decrease energy expenses through careful targeting of the food matrices [22]. In spite of the investment needs in PEF technology, using thermal treatments in combination with a PEF has been suggested as a strategy to increase its effectiveness and reduce energy costs [24]. 

The impact of the PEF on microorganisms and milk enzymes can be improved by combining it with other technological methods, such as mild heating, i.e., operating below conventional pasteurization temperatures, acting as complementary to PEF treatment. Although the effects of the PEF on milk and dairy product processing have been recently studied, the specific processing conditions are extensively discussed by several authors, focusing on evaluating the combined impact of these techniques [25].

Concerning milk pasteurization, the PEF is an emerging alternative method studied in bovine milk, including skim and whole milk [26,27,28,29]. However, there are limited published studies with goat milk [30,31,32,33]. Pasteurized goat’s milk is not as commonly found in the market as cow’s milk. However, improvements in the pasteurization of goat milk, especially with processes that allow the maintenance of its nutritional qualities, will certainly have an impact on its availability in the market [32].

Sharma et al. [18] also reported that PEF-treated raw bovine whole milk was microbiologically stable for 21 days at 4 °C and similar to thermally treated milk (63 °C for 30 min or 73 °C for 15 s).

Beyond the inactivation capacity of the PEF treatment, another advantage is the fact that this type of treatment does not have significant repercussions on the physical properties such as pH, color, or particle size distribution, as demonstrated by studies with a PEF treatment of commercial low-fat bovine milk at 10 kV·cm^−1^ for 30 μs [16].

The objective of the present work was to evaluate the effectiveness of microbial inactivation of *Listeria monocytogenes* of the PEF-processed goat milk in combination with mild temperature pasteurization and reduce the energy requirements of this step.

## 2. Materials and Methods

In the following sections, milk samples pasteurized with heat treatment only are referred to as “HT” samples and samples treated with a combination of pulsed electric fields and heat treatment are referred to as “PEF + HT”.

### 2.1. Milk Samples

UHT cow’s milk (1.5% milk fat) was obtained from a retail store, and goat milk was kindly provided by a local dairy farm of traditional cheese in Melgaço (Prados de Melgaço, Melgaço, Portugal). Samples of raw goat milk were obtained in the morning within 2 h after milking, collected in low-density polyethylene (LDPE) glass bottles, and transported at 4 ± 1 °C, directly to the IPVC Food Processing and Engineering Laboratory, Viana do Castelo, Portugal. All samples were kept at 4 ± 1 °C and used within a maximum of 12 h for their respective treatments and analysis, all of which were carried out in triplicate.

### 2.2. Regeneration of Bacterial Cultures

Cultures of *E. coli* (ATCC 11775) and *L. monocytogenes* ATCC 13932, preserved at −80 °C in a BHI medium with 15% (*v*/*v*) glycerol (Merck, Darmstadt, Germany) were used in this work. The regeneration of *E.coli* and *L. monocytogenes* was performed in 5 mL of a brain–heart infusion medium, BHI (Biokar Diagnostics, Beauvais, France), at 37 °C overnight.

### 2.3. Preparation of the Inoculum for Use in Inactivation Studies

Before the use in inactivation studies, microbial cultures were diluted at 1:100 with the respective culture medium and grown at 30 °C until the exponential growth phase was reached. The concentration of the inoculum was adjusted via densitometry until a 0.5 MacFarland suspension was obtained (Biosan DEN-1B, Riga, Latvia) prepared by the direct suspension of four or five morphologically similar colonies, isolated in a TSA medium in a sterile 0.85% (*w/v*) NaCl solution until turbidity of 0.5 MacFarland was obtained (Biosan DEN-1B, Riga, Latvia).

### 2.4. Pulsed Electric Field Tests

For non-thermal processing of inoculated samples, PEF equipment was used—EPULSUS^®^-LPM1A-10 (EnergyPulse Systems, Lda., Lisbon, Portugal) for laboratory scale. This generator produces positive, unipolar rectangular pulses and is equipped with a continuous mode over a collinear treatment chamber with an internal diameter of 1.0 cm and a gap distance of 1.0 cm between the metal (titanium) electrodes. Samples were processed with a PEF in continuous mode, using a fixed electric field of 10 kV·cm^−1^. The temperature was measured at the entrance and exit of the PEF treatment assembly. Prior to each test, the treatment line was cleaned with distilled water, sanitized by pumping a 70% ethanol solution through it, and then rinsed with sterile distilled water. A peristaltic pump (Watson Marlow 313S, Marlow, United Kingdom) was used to pump the microbial suspension through the system at a flow rate of 2.92 L·h^−1^. This flow rate was decided after preliminary studies that showed that, under lab conditions, it provided a maximum reduction in the viability of microbial cells. After treatment, the temperature of the treated product was measured, and 5 mL was collected from sterile test tubes, which were immediately placed on ice until the quantification of surviving cells was performed. All tests were performed in triplicate.

### 2.5. Combined PEF and Milk Thermal Treatments

Inoculated raw goat milk and UHT cow’s milk kept at 4 °C were pre-treated using a low-intensity PEF followed by heat treatments (PEF + HT). PEF conditions were: constant pulse width of 50 µs, frequency of 3 Hz, an electric field strength of 10 kV·cm^−1,^ and a flow rate of 2.92 L·h^−1^. Thermal treatments were then carried out in a heat exchanger unit FT74XTS HTST/UHT system (Armfield, UK) at a constant flow rate of 10 L·h^−1^, using temperatures in the range of 63–75 °C (63, 66, 69, 72, and 75 °C), and a holding tube of 2 s.

Samples of goat and cow’s milk subjected only to thermal treatment (HT) were used as controls.

In this combined heat thermal treatment with a PEF, a continuous mode of the PEF was performed using laboratory-scale PEF equipment as described before, with positive rectangular pulses before pasteurization and using only this sequence. Immediately after processing, HT or PEF + HT milk samples were collected in duplicates under sterile conditions and immediately placed in an ice bath until further analysis.

The electrical conductivity of fresh milk at 4 ± 1 °C was measured using a pH-Conductivity Meter Orion 4-Star (Thermo Scientific, Waltham, MA, USA).

### 2.6. Cleaning-in-Place of Heat Exchanger Unit

The product lines of the heat exchanger were cleaned and sanitized before and after each experiment using a cleaning-in-place procedure involving pre-rinsing, chemical cleaning, disinfection, and final rinsing. Cleaning and sanitation were carried out using distilled water (60 °C in a heat-exchange unit) followed by a 5% (*w*/*v*) NaOH solution, sterilized distilled water, a 1% (*v*/*v*) HNO_3_ solution, and finally sterilized distilled water. 

### 2.7. Quantification of the Surviving Cells

After spiking the cow’s and goat’s milk with the test microorganism, a sample was taken immediately before the treatment (HT or PEF + HT) to determine the initial concentration of cells. After treatment, a sample was immediately cooled on ice. Enumeration of the surviving cells was performed after decimal dilutions in a maximum recovery diluent (Liofilchem srl, Roseto degli Abruzzi, Italy) and quantified according to ISO 16649-2:2001 by incorporation into a TBX medium for counting *E. coli* after incubation at 37 °C for 24 h, and counting *L. monocytogenes* was performed vis inoculation on the surface of a Listeria chromogenic agar base, ALOA (Biomerieux, France), after incubation at 37 °C for 48 h, as described on ISO 11290-1:2017. The mean values of viable counts and respective standard deviations were estimated and expressed as the logarithm of colony-forming units per mL of product, i.e., log (CFU·mL^−1^).

Calculations of thermal death kinetic parameters, namely decimal reduction time (*D*), were estimated using the log-linear equation

(1) $$log\left(N\right)=log\left(N_{0}\right)-\left(\frac{t}{D}\right)$$

where *N*_0_ and *N* are, respectively, the initial population and number of survivors after the treatment. The *z* value was calculated according to:

(2) $$z=\frac{T_{2}-T_{1}}{log\left(D_{1}\right)-log\left(D_{2}\right)}$$

Sub-lethally injured cells were quantified using plating cells after milk treatment, in parallel on TSAYE and TSAYE with 5% NaCl [34].

### 2.8. Measurements of pH, Electrical Conductivity, Titratable Acidity (TA), Total Soluble Solids (TSS), and Viscosity in Goat Milk

The pH, *TA*, and *TSS* of HT and PEF + HT samples were measured at room temperature (20 ± 2 °C) according to the AOAC standard method 981.12, 947.05, and 932.12, respectively [35].

Milk viscosity evaluation was carried out using a Thermo Haake rotational viscometer (model VT 550) with concentric cylinders (NV ST 807-0713 CE and NV 807-0702) and collected using the software program Pro RheoWin (version 2.93, Haake). Flow behavior was evaluated according to a previously described method [35]. Flow curves were generated using a shear rate increased from 10.82 s^−1^ to 221.80 s^−1^ in the first 2 min, under a controlled temperature of 20.0 ± 0.1 °C, through water circulation in a temperature-controlled bath (Thermo Haake K20) coupled to the equipment. Regarding the physico-chemical characterization, five milk samples collected at the Prados de Melgaço facilities (March 29th and April 4th, 6th, 11th, and 13th) were used. Results are presented as the average of all determinations for each parameter. All the values were obtained in triplicate.

### 2.9. Heat Treatment and PEF Energy Calculations

Heating energy or sensitive heat, *Q*, expressed in kJ∙h^−1^, during thermal treatments was calculated according to the temperature increase (∆*T*) promoted by the heat exchanger after the PEF treatment. The heat-energy needs were calculated according to other similar studies using Equation (3) [24,27,36].

*Q* = *m·Cp·*∆*T* (3)

where *m* is the mass rate (m^3^∙h^−1^) of heat-treated milk and *Cp* (kJ/(kg∙K)) is the specific heat of milk.

The mass rate of vapor, *m_v_* (kg∙h^−1^), needed for PEF + HT pasteurization (63 °C) and thermal pasteurization (HT at 72 °C) was calculated using Equation (4).

*Qv* = *m_v_·H_v_* (4)

where *Qv* (kJ∙h^−1^) vapor heat energy (kJ∙h^−1^) and *H_v_* is the energy of 1 kg of steam (kJ), obtained from saturated steam property tables, considering a steam temperature of 70 °C in the case of PEF + HT treatment at 63 °C, and 80 °C in the case of HT treatment at 72 °C.

The energy delivered using the PEF equipment, *Q_PEF_* (kJ∙L^−1^), was calculated according to Sampedro, 2014 [37] using Equation (5).

(5) $$Q_{PEF}=\frac{f\cdot Q_{pulse}}{m}$$

where *f* is the pulse frequency (Hz), *Q_pulse_* is the energy per pulse (J), and *m* is the flow rate of milk (L∙s^−1^) entering the PEF equipment system.

For the PEF + HT treatment, the total required energy, *Q_T_*, was then calculated using Equation (6).

*Q_T_* = *Q_PEF_* + *Q* (6)

### 2.10. Data Analysis

For all treatments, statistical differences were determined using data from three different batches of milk (*n* = 3) and analyzed using analysis of variance (one-way ANOVA) with a significance level of α = 0.05. Tukey HSD tests were used to evaluate differences following significative ANOVAs. All statistical analyses were performed using the R software (R version 4.3.1, 2023, R Foundation for Statistical Computing). All graphs were produced using the same software.

## 3. Results and Discussion

Alternative pasteurization methods, which avoid the exposure of milk to high temperatures but achieve the needed reduction of the microbial load, are of great interest. In this sense, the PEF has been used in several food products with success in microbial inactivation.

### 3.1. Microbial Inactivation

The effectiveness of PEF treatments on inactivating bacteria is highly influenced by the size of the cell. As the cell size decreases, the resistance to PEF treatments increases due to the smaller transmembrane potential created by the external electric field [21,38], which is probably one of the reasons why *L. monocytognes* is a bacterium with high resistance to PEF treatments [38]. The fact that it is a pathogen and a common contaminant of dairy products, as well as its resistance to heat treatments, determined its choice for the inactivation tests that were conducted on goat’s milk, with the goal of achieving a comparable reduction of 5 log cycles in both types of treatment (with and without PEF). This is the minimum reduction for *L. monocytogenes* as recommended by the USFDA’s “Control of *Listeria monocytogenes* in Ready-To-Eat Foods: Guidance for Industry” [39] and its resistance is supported by several previous works on the bacterium’s thermotolerance (e.g., [40]) and thermal resistance in goat’s milk [41].

**Figure 1:**
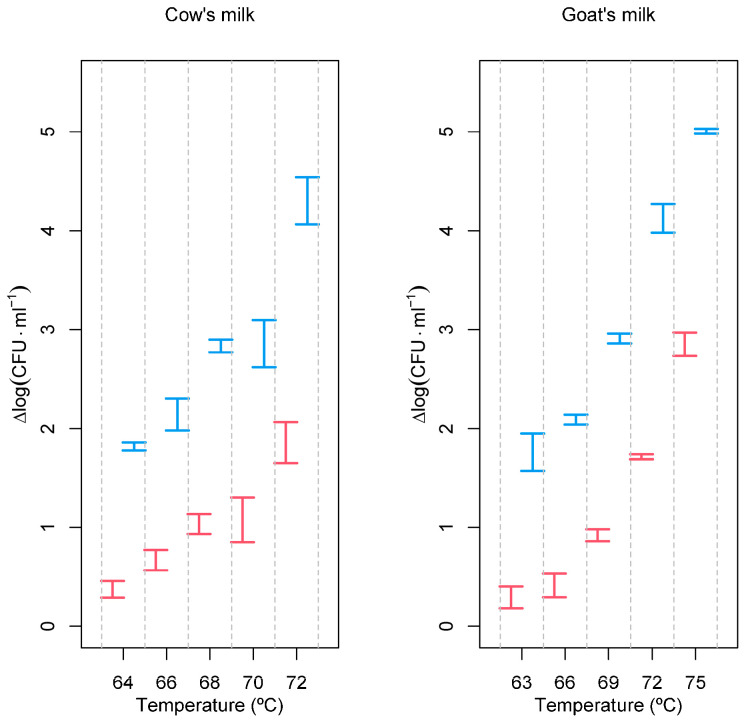
Inactivation of *L monocytogenes* as a function of processing conditions. Red—heat treatment only; blue—PEF combined with heat treatment. Experimental temperatures ranged between 63 and 75 °C. Inactivation, expressed as Δlog (CFU·mL^−1^), was calculated according to log (*N*_0_/*N*), where *N*_0_ is the initial number of cells and *N* is the number of cells after the treatment.

Tests were performed at different temperatures with and without the PEF in cow’s and raw goat’s milk. The milks were inoculated with a *L. monocytogenes* culture to obtain an initial concentration of at least 8 log (CFU·mL^−1^), and the reduction in the number of viable cells was determined after a fixed treatment time of 2 s (Figure 1). 

As expected, Figure 1 clearly shows that the inactivation of *L. monocytogenes* increases just by increasing temperature. But, this figure also shows that inactivation is much more evident and more pronounced when the PEF is also used as a pre-treatment, and this is true for both cow’s and goat’s milk. These results are so evident that analysis of variance to compare the effect of temperature, coupled or not with the PEF, would be unnecessary. However, ANOVAs are presented because they facilitate post hoc Tukey HSD tests, comparing all pairs of processing conditions, i.e., all pairs of temperatures with and without PEF. As these involve a total of 45 comparisons, but only a few relevant for discussion are shown in Table 1 and Table 2.

Table 1, referring to cow’s milk, highlights the fact that the maximum temperature used (72 °C, with no PEF) has the same effect over *L. monocytogenes* as the minimum temperature condition used with the PEF (PEF + 64 °C). After survival counting, calculations of the decimal reduction time (*D*) allowed the calculation of the *z* values for both types of treatments (Table 3).

The application of the PEF alone did not allow for a significant reduction in the *L. monocytogenes* load (well below the 5 log cycles reduction), and this was why cheeses were not produced with milk only treated with the PEF (as the reduction of viable cells is too low to be considered pasteurization). However, there is a significant number of cells affected by the PEF even though they are not dead: the log reduction after PEF treatment and plating on a medium with and without 5% NaCl showed a significant increase in the number of affected cells (lethal and sub-lethal injured), 3.351 ± 0.008 log (CFU∙mL^−1^) and 1.765 ± 0.083 log (CFU∙mL^−1^) reduction, respectively, meaning that after the PEF, 97.4% of survivor *L. monocytogenes* cells were sub-lethally injured, which is probably why a high efficiency of reducing the population of viable cells using the combined treatment was achieved. Most studies on microbial inactivation induced using the PEF use high electric field strengths (20–50 kV∙cm^−1^), while low electric field strengths tend to cause sub-lethal injuries to cells. Man-Sheng Wang et al. [42] observed that after a PEF treatment with field strengths of 5–10 kV·cm^−1^, about 90% of the cells were sub-lethally injured. Several studies have demonstrated the occurrence of a significant number of sub-lethally damaged cells after PEF treatments, and the extent of this damage is influenced by the type of bacteria, the pH of the treatment medium, the duration of treatment, and the strength of the electric field applied [34].

The PEF pre-treatment allowed for a significant increase in the log reduction of the *L. monocytogenes* population (Figure 1). In goat’s milk, after 2 s of holding time at 66 °C, a reduction of 0.408 ± 0.121 log (CFU∙mL^−1^) of the *L. monocytogenes* population was obtained. If pre-treated with the PEF, a reduction of 2.084 ± 0.049 log (CFU∙mL^−1^) was achieved, corresponding to a very significant increase in the efficacy of the milk processing. The effect of the PEF treatment on cow’s milk was not so pronounced, even though it managed to increase the reduction in viable cells from 0.679 ± 0,10 log (CFU∙mL^−1^) at 64 °C to 2.125 ± 0.16 log (CFU∙mL^−1^) at 72 °C.

In goat’s milk, the combined use of the PEF and mild temperature allowed us to obtain a 5.002 ± 0.029 log (CFU∙mL^−1^) reduction after the PEF treatment combined with a heat treatment for 2 s at 75 °C. In the absence of the PEF pre-treatment, the reduction obtained was just 2.858 ± 0.117 log (CFU∙mL^−1^).

Both for cow’s and goat’s milk, the increase in the temperature needed to decrease the decimal reduction time is much higher in the PEF pre-treated milk than in milk just subjected to thermal processing (Table 3).

In this study, *D* values are within the same order of magnitude as values already published, although there is some variation from work to work, depending significantly on how the experimental data were obtained, the type of treatment (continuous or in batch), and the strain used. For example, in cow’s milk, a *D*_65_ value of 0.1 min for *L. monocytogenes* has been reported [43,44]. This is exactly the value obtained for the same microorganism but in goat’s milk. In cow’s milk, its sensitivity to temperature proved to be higher, with a *D*_65_ value of 3.89 s. The resistance of *L. monocytogenes* in cow’s and goat’s milk proved slightly different. The sensitivity of *L. monocytogenes* to high temperatures is lower in goat’s milk than in cow’s milk, as revealed by the *D* calculated. This is particularly visible for lower temperatures (e.g., 63 °C), where *D*_63_ in goat’s milk is 28% higher than in goat’s milk but only 12.8% higher at 72 °C. The influence of the PEF on lethality is more notorious when combined with lower temperatures and in goat’s milk. At 63 °C, there is a reduction of 83.6% in the decimal reduction time when the PEF is used as a pre-treatment, revealing how efficient this step might be in decreasing the temperature of milk processing.

The PEF pre-treatment significantly increases the *z* values obtained for the inactivation of *L. monocytogenes* in cows’ and goat’s milk. In fact, in cows’ milk, the increase in temperature needed to decrease the decimal reduction time by a fraction of 10 is 76.4% higher when the milk is pre-treated with the PEF than when milk is only heat processed. In goats’ milk, this effect is even more pronounced as the estimated increase is 112.8%. This suggests that PEF may induce some heat resistance in the population of cells that are not dead or injured by this treatment. Previous works on the resistance of *L. monocytogenes* to a PEF have also suggested that the PEF might influence the levels of expression of chaperone proteins, impacting the resistance of the microorganism to mild heat treatments and heat resistance [45].

**Figure 2:**
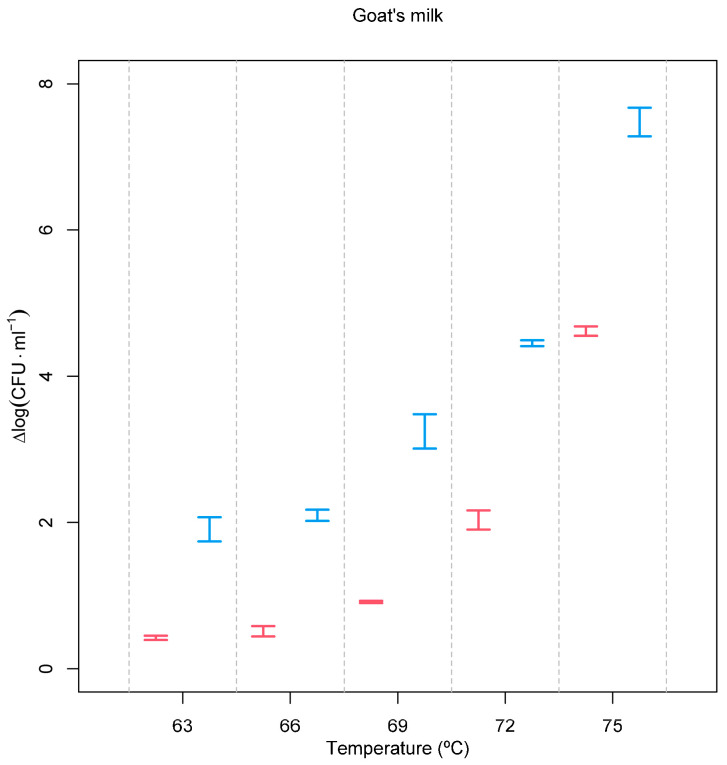
Inactivation of *E. coli* as a function of processing conditions. Red = heat treatment only; blue = heat treatment combined with the PEF. Experimental temperatures ranged between 63 °C and 75 °C. Inactivation, expressed as Δlog (CFU·mL^−1^), was calculated according to log (*N*_0_/*N*), where *N*_0_ is the initial number of cells and *N* is the number of cells after the treatment.

Applying the same treatment conditions to goat’s milk spiked with a common bacterium usually associated with poor hygiene practices, *E. coli* allowed us to observe its expected lower resistance to both treatments (Figure 2) and an even higher effect of the pre-treatment with the PEF. At 75 °C, a 4.617 ± 0.066 log (CFU·mL^−1^) reduction was observed, but this value was increased to 7.498 ± 0.198 log (CFU·mL^−1^) when milk was pre-treated with a PEF. A recent article showed that it is possible to achieve a reduction of 3.87 log (CFU∙mL^−1^) of *E. coli* at 40 kV·cm^−1^ and 13 ms. However, this is a Gram-negative bacteria with little resistance to pulsed electric fields, and a high electric field strength was used [33].

**Figure 3:**
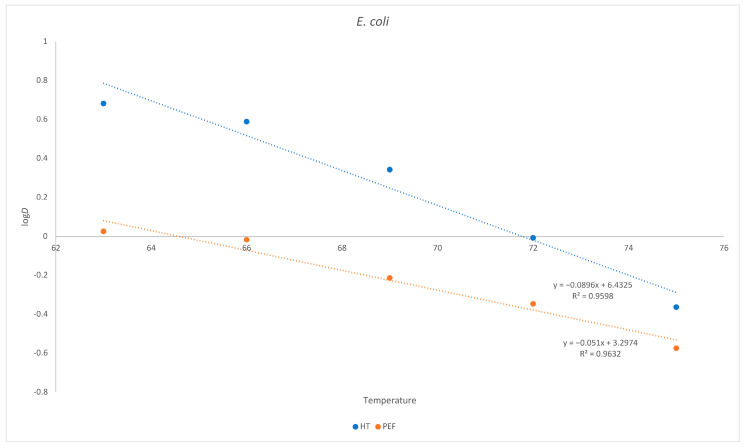
Inactivation of *E. coli* as a function of temperature in goat´s milk. Influence of temperature on *D*. Red = heat treatment only; blue = heat treatment combined with PEF.

As already demonstrated for *L. monocytogenes*, the same holds true for *E. coli.* Despite being more sensitive to thermal death after a PEF treatment, the requirement for a greater temperature increase to reduce the *D* value is higher when the PEF is applied (Figure 3).

### 3.2. Chemical Analysis

During the experiments with raw goat’s milk, the following chemical parameters were monitored: conductivity, pH, *TA*, *TSS*, fat, protein, dry extract (defatted), urea, and lactose, according to the results of Table 4, to ensure the analytical control of raw milk.

The results in Table 4 demonstrate that the samples of raw goat milk are quite homogeneous over the collected samples, as observed by the low standard deviation values obtained for all the parameters analyzed.

At the same time, some physical parameters of goat´s milk were also analyzed after thermal treatments to evaluate the effect of temperature on the final characteristics.

Table 5 highlights the results of pH, electrical conductivity, titratable acidity, and *TSS* after thermal treatments (HT and PEF + HT).

The average pH values (6.76 ± 0.03) and total soluble solids (10.70 ± 0.26) in raw milk did not change after the treatment. The titratable acidity decreased from a maximum of 0.150 ± 0.003% (% in lactic acid) in raw milk to values below 0.136% for both pasteurized and combined treatment samples. Conductivity decreased as a function of the increase in temperature for both treatments.

Regarding the viscosity values, they did not differ between treated and untreated samples, with goat’s milk showing a non-Newtonian fluid behavior. The viscosity decreases with increasing shear rate, showing a pseudoplastic behavior in all samples analyzed.

### 3.3. Energy Considerations

Considering the equipment used in this study, the energy per unit time (kJ∙h^−1^) needed for the HT stage, with and without the PEF pre-treatment (*Q_PEF+HT_* and *Q_HT_*, respectively), was calculated. In this case, the milk flow rate of the heat exchanger was 10 L∙h^−1^. To determine mass rate, it was assumed that goat and cow milk density was 1007.245 kg∙m^−3^ (*T* = 40 °C) and 1030.8 kg∙m^−3^ (*T* = 50 °C), respectively. The *Cp* values considered for goat’s milk and cow’s milk were 3.79 kJ∙(kg∙K)^−1^ and3.77 kJ∙(kg∙K)^−1^, respectively [30,46,47,48,49]. Table 6 summarizes the energy needed to raise milk temperatures in the thermal step of the studied processing conditions.

It can be observed that the energy requirement by the heat treatment stage without the PEF is much higher. In this study, this means almost three times less energy requirements in the heat exchanger at a milk flow rate of 10 L∙h^−1^. Similar conclusions have been reported by Sharma et al. [27]. The economic advantage in industrial applications can be much more significant as the need for thermal energy for the thermal treatment with the PEF as a pre-treatment can be reduced to a great extent. These needs depend on several factors, with a special reference to the efficiencies of heat exchangers and the steam supply equipment, noting, e.g., that boilers in industrial plants are major energy consumers. The results obtained in this study with laboratory-scale equipment revealed that energy conservation can reach 50%. The steam flow rate (*m_v_*) required to complete the pasteurization operation using PEF + HT at 63 °C is 0.33 kg·h^−1^, which is three times lower than that required using the HT treatment at 75 °C (approx. 0.97 kg∙h^−1^), assuring a similar decimal reduction in the number of viable microorganisms.

Moreover, considering the total energy requirement for the PEF operation, it can be observed that it has no great expression compared to the steam supply. The value of *Q_PEF_* was determined, taking into consideration the equipment and the PEF conditions, which represents 159.8 kJ·L^−1^ of treated milk, amounting to approximately 466.6 kJ∙h^−1^. In Table 3, this value was added to *Q_PEF + HT_* to calculate *Q_Total_*. As expected, the latter is much lower than in the case of the HT treatment.

Despite equipment investment, many authors report that they can be quickly recovered since the installation cost is negligible and the production of steam needed for the heat treatments will be significantly lower [37]. Thus, PEF + HT treatment has a much lower environmental impact than conventional thermal pasteurization [19,22,23,24,25].

## 4. Conclusions

The results obtained as the outcome of this research provide useful information to dairy industries, especially those seeking technologies with the potential to increase energy efficiency. In this sense, pulsed electric fields (PEFs) show great potential as a pasteurization method for goat’s milk and cheese processing.

In this work, *L. monocytogenes*, because of its high resistance and predominance in dairy products, was used to evaluate the impact of PEF treatments on reducing the number of viable bacterial cells in cow’s milk and mainly in goat’s milk. It was observed that there was a similar reduction in the number of viable *L. monocytogenes* cells when using a typical 72 °C pasteurization or a 64 °C pasteurization with a PEF pre-treatment. The use of the PEF, along with a mild temperature (2 s at 64 °C), enabled us to obtain a 5 log (CFU∙mL^−1^) reduction of viable cells in comparison to the 2.9 log (CFU∙mL^−1^) obtained without the PEF pre-treatment.

*E. coli* was also included in this study because it serves as a general hygiene indicator. It was observed that the effect of the pre-treatment with the PEF on this bacterial species was even more noticeable, with a reduction of 7.5 log (CFU∙mL^−1^) of viable cells, while a reduction of only 4.6 ± 0.198 log (CFU∙mL^−1^) was obtained without the PEF pre-treatment.

The combined application of the PEF and mild heating demonstrated its effectiveness in inactivating *L. monocytogenes* and *E. coli* in goat’s milk, serving as an interesting alternative to traditional heat treatment within the dairy industry. Concerning energy conservation, it has been confirmed that employing the PEF as a pre-heating process significantly reduces the heating energy requirements in thermal treatment by approximately three times. This, in turn, results in substantial water vapor conservation and subsequently reduces the workload and costs associated with boilers. Furthermore, utilizing PEF technology is not only cost-effective but also enhances the preservation of product quality attributes. Despite the potential of the PEF as an alternative to high temperature, its influence on the nutritional and functional quality of goat’s milk, as well as on the sensory quality and consumer acceptability, still needs to be clarified.

## Figures and Tables

**Table 1:** ANOVA table reporting the combined effects of five levels of heat treatment alone and combined with the PEF applied to cow’s milk. The results of three *post hoc* Tukey HSD tests related to 64 and 72 °C, with and without the PEF, are also shown.

	DF	SS	MS	F Value	Pr (>F)
Treatment	9	38.97	4.330	147.4	2.73 × 10^−16^
Residuals	20	0.59	0.029		
	Tukey HSD (95% confidence level)				
		diff	lwr	upr	*p* adj
64 PEF + HT-64 HT		1.430	0.935	1.926	0.000
72 HT-64 PEF + HT		0.026	−0.470	0.522	1.000
72 PEF + HT-72 HT		2.496	2.000	2.991	0.000

**Table 2:** ANOVA table reporting the combined effects of five levels of heat treatment alone and combined with the PEF applied to goat’s milk. The results of four post hoc Tukey HSD tests related to 63, 69, 72, and 75 °C, with and without the PEF, are also shown.

	DF	SS	MS	F Value	Pr (>F)
Treatment	9	63.67	7.074	627	<2 × 10^−16^
Residuals	20	0.23	0.011		
	Tukey HSD (95% confidence level)				
		diff	lwr	upr	*p* adj
63 PEF + HT–63 HT		1.450	1.143	1.757	0.000
72 HT–63 PEF + HT		−0.043	−0.350	0.264	0.999
75 HT–69 HT		−0.053	−0.360	0.254	0.999
75 PEF + HT–75 HT		2.143	1.836	2.450	0.000

**Table 3:** Thermal death behavior of *L. monocytogenes* in cow’s and goat´s milk.

Milk	Processing	*Log* (*D*) = *f* (*T*)	*z* Value
Cow	HT	$log\left(D_{r}\right)=-0.0789\;T_{r}+5.7179$ R^2^ = 0.9609	12.7 °C
Cow	PEF + HT	$log\left(D_{r}\right)=-0.0446\;T_{r}+2.9035$ R^2^ = 0.9442	22.4 °C
Goat	HT	$log\left(D_{r}\right)=-0.0854\;T_{r}+6.238$ R^2^ = 0.9841	11.7 °C
Goat	PEF + HT	$log\left(D_{r}\right)=-0.0401\;T_{r}+2.5998$ R^2^ = 0.9877	24.9 °C

**Table 4:** Chemical parameters of raw goat’s milk (mean ± standard deviation).

pH	Conductivity (mS/cm)	*TA* (% Lactic Acid)	*TSS* (°Bx)	Fat (%)	Protein (%)	Dry Extract (%)	Urea (mg/L)	Lactose (%)
6.76	5.47	0.15	10.70	4.87	3,48	8.80	623.8	4.72
±0.029	±0.061	±0.003	±0.265	±0.720	±0.178	±0.180	±23.787	±0.021

**Table 5:** Physico-chemical parameters of raw goat’s milk and after thermal treatment (all values expressed as mean ± standard deviation).

Milk Samples		pH	Electrical Conductivity (mS·cm^−1^)	*TA*	*TSS* (°Bx)
				(% Lactic Acid)	
Raw milk		6.76 ± 0.03	5.47 ± 0.06	0.150 ± 0.003	10.70 ± 0.26
HT	63	6.69 ± 0.02	5.20 ± 0.03	0.134 ± 0.004	10.57 ± 0,32
	66	6.68 ± 0.05	4.80 ± 0.03	0.134 ± 0.005	10.20 ± 0.61
	69	6.67 ± 0.04	4.57 ± 0.02	0.136 ± 0.004	10.60 ± 0.20
	72	6.72 ± 0.04	4.07 ± 0.06	0.133 ± 0.003	10.57 ± 0.12
	75	6.68 ± 0.02	3.34 ± 0.02	0.135 ± 0.003	10.17 ± 0.25
PEF + HT	63	6.67 ± 0.04	5.24 ± 0.03	0.134 ± 0.002	10.70 ± 0.26
	66	6.67 ± 0.03	4.71 ± 0.02	0.133 ± 0.004	10.40 ± 0.20
	69	6.66 ± 0.02	4.45 ± 0.05	0.133 ± 0.003	10.13 ± 0.25
	72	6.70 ± 0.03	3.97 ± 0.04	0.135 ± 0.003	10.73 ± 0.21
	75	6.65 ± 0.02	3.45 ± 0.03	0.136 ± 0.001	10.30 ± 0.17

**Table 6:** Sensitive heat necessary to raise milk temperature and complete pasteurization. *Q_T_* = *Q_PEF_* + *Q_PH_*.

	*T* (°C)	*Q_PEF+HT_* (kJ∙h^−1^)	*Q_T_* (kJ∙h^−1^)	*Q_HT_* (kJ∙h^−1^)
Goat’s milk	63	878.015	1344.615	2214.126
	66	992.539	1459.139	232.650
	69	1107.063	1573.663	2443.173
	72	1221.587	1688.187	2557.697
	75	1336.110	1802.710	2672.221
Cow’s milk	63	893.807	1360.407	2253.947
	66	1010.390	1476.990	2370.531
	68	1088.112	1554.712	2448.253
	70	1165.835	1632.435	2525.975
	75	1360.141	1826.741	2720.281

## Data Availability

The data used to support the findings of this study can be made available by the corresponding author upon request.
